# Magnetic genes: Studying the genetics of biomineralization in magnetotactic bacteria

**DOI:** 10.1371/journal.pgen.1008499

**Published:** 2020-02-13

**Authors:** Hayley C. McCausland, Arash Komeili

**Affiliations:** 1 Department of Molecular and Cell Biology, University of California, Berkeley, Berkeley, California, United States of America; 2 Department of Plant and Microbial Biology, University of California, Berkeley, Berkeley, California, United States of America; Naval Research Laboratory, UNITED STATES

## Abstract

Many species of bacteria can manufacture materials on a finer scale than those that are synthetically made. These products are often produced within intracellular compartments that bear many hallmarks of eukaryotic organelles. One unique and elegant group of organisms is at the forefront of studies into the mechanisms of organelle formation and biomineralization. Magnetotactic bacteria (MTB) produce organelles called magnetosomes that contain nanocrystals of magnetic material, and understanding the molecular mechanisms behind magnetosome formation and biomineralization is a rich area of study. In this Review, we focus on the genetics behind the formation of magnetosomes and biomineralization. We cover the history of genetic discoveries in MTB and key insights that have been found in recent years and provide a perspective on the future of genetic studies in MTB.

## Author summary

Open any biology textbook and you are likely to learn that bacteria—unlike the cells of plants, animals, and other eukaryotes—do not contain organelles to compartmentalize and facilitate cellular functions. However, over the past several decades, many different bacterial organelles have been discovered. In this Review, we highlight magnetotactic bacteria (MTB), which are a group of organisms capable of producing organelles called magnetosomes where nano-sized crystals of magnetic material are synthesized and housed. In order to understand how and why MTB form magnetosomes, it is important to study the genes involved. Here, we lay out the history of genetic studies in MTB and more recent discoveries about which genes are involved at each step in the process of magnetosome formation and discuss where the field is headed.

## Introduction

Bacteria, according to the canonical definition, do not have subcellular compartments for organization or specialized functions. Yet microbiologists are becoming increasingly aware that many bacteria do have organelles, some of which are capable of manufacturing biomaterials with specialized functions [1]. MTB present a particularly elegant example of the biological behaviors that are mediated by intracellular compartments [2]. MTB are a group of bacteria spanning multiple phyla that can be found in aquatic environments all over the world [3–5], where they inhabit low oxygen environments, and are most often found at the oxic–anoxic interface in the water column or sediments [6]. MTB are characterized by their ability to form organelles called magnetosomes—lipid-bounded compartments in which biomineralization of magnetic crystals of magnetite (Fe_3_O_4_) and/or greigite (Fe_3_S_4_) occurs [3]. Magnetosomes align in one or multiple chains along the cell, creating a magnetic dipole that allows MTB to passively align along Earth’s magnetic field lines [7]. This is thought to help MTB perform more efficient chemotaxis and aerotaxis in the water column as their swimming behavior is restricted to one dimension instead of a three dimensional run and tumble search: a process called magneto-aerotaxis [6]. The regulated process of biomineralization has made MTB an attractive area of study in basic and applied biology, geochemistry, and physics alike. A greater understanding of the molecular processes needed to form magnetosomes will enhance studies in each of these fields, as knowledge of what is occurring on a molecular scale can lend greater precision to technical applications and provide systems-level information for studying the large-scale impacts of MTB.

The ecological impact of MTB is one rising area of research in the field. In recent years, our understanding of the diversity of MTB has expanded greatly. In the process of biomineralization of magnetite or greigite, MTB take up large amounts of dissolved iron from the surrounding environment and sequester it in magnetosomes as iron crystals. As such, the role that MTB play in iron cycling in both freshwater bodies and the ocean is potentially quite large [5,8]. Conservative estimates from Amor and colleagues indicate that estuarine and oceanic MTB may take up anywhere from approximately 1% to 50% of dissolved iron inputs (approximately 9 × 10^8^ kg per year) in their environments [8]. Having a greater understanding of the iron regulation strategies encoded within the genomes of MTB, as well as how iron is taken up and distributed to magnetosomes, is important for a more accurate picture of the role of MTB in their aquatic environments.

In this same vein, fossils of magnetosomes can help us understand the environmental conditions that were present when MTB originated and the evolution of life on Earth. Magnetofossils—currently dating back to approximately 1.9 Ga—may reflect changes that occurred in sediments and the water column and have the potential to serve as an indicator of redox and oxygen levels in ancient environments [9]. Based on phylogenetic analyses, the origins of biomineralization may have occurred much earlier, in the mid-Archaean (approximately 3 Ga), when the ability to biomineralize may have provided an advantage in coping with reactive oxygen species, avoiding harmful UV radiation, and/or navigating ferrous-iron gradients [10,11]. Understanding the genetic factors behind the formation of magnetosomes that are common across modern MTB can provide insight into the conditions and processes that were present when the first MTB originated [12]. Genetic analysis may also uncover unknown functions of magnetosomes and can hint at the conditions that were needed to produce ancient magnetosomes.

Additionally, the use of MTB in various biotechnological applications is promising. Magnetosomes are currently being developed for use as magnetic resonance imaging (MRI) contrast agents, drug delivery systems, hyperthermic and photothermic treatments for cancer, bioremediation of heavy metals, and other nanotechnologies [13–15]. In order to efficiently produce the large numbers of magnetosomes required in these applications, it is critical to understand how magnetosomes are produced.

**Fig 1:**
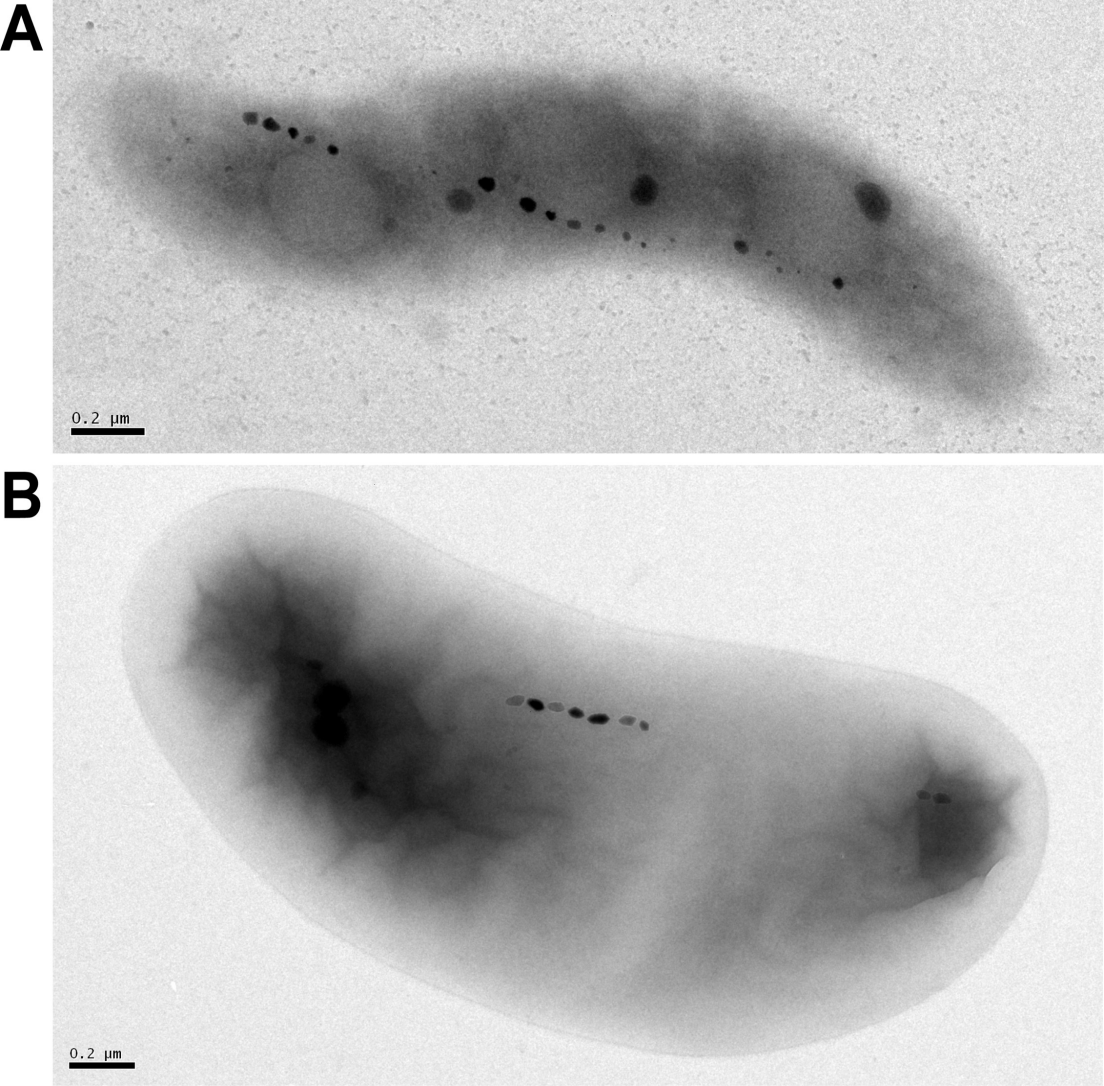
The MTB model systems. (A) TEM image of wild-type AMB-1 cell. (B) TEM image of wild-type RS-1 cell, scale bar 200 nm. Reprinted with permission from Rahn-Lee and colleagues. [21]. TEM, transmission electron microscopy.

Taking up iron from the environment for biomineralization, producing phospholipid membranes of a specific size, and aligning magnetosomes in a chain is a complex, tightly controlled process—one that is interesting in itself but also provides more general insights into the precise formation of organelles. MTB encode the genes necessary for these processes in magnetosome gene clusters (MGCs) [16,17]. The MGCs in the most well-studied model organisms—*Magnetospirillum magneticum* AMB-1, *M*. *gryphiswaldense* MSR-1, and *Desulfovibrio magneticus* RS-1 (Fig 1)—are structured as magnetosome gene islands (MAIs). In both AMB-1 and RS-1, the MAI is defined by repeat regions on either side of the large chromosomal region [18,19]. Across related species, there is a large amount of genetic homology in the MAI [20]. The functions of many of the genes within MGCs have been investigated, but much remains unknown in each of the model organisms, and there is even more to be discovered about other species and phyla of MTB.

In this Review, we lay out the history of landmark genetic discoveries in revealing important insights into the process of magnetosome and biomineral formation by MTB. We also dive into more nuanced views of genetics that have come out in recent years in step with advances in genetic techniques. Finally, we will address the next directions that the field is taking to learn more about the genetics of MTB.

## Genetics in *Magnetospirilla*

The discovery of MTB [7] fueled broad interest in understanding and exploiting the process of organelle formation and biomineralization. Development of genetic systems greatly accelerated the discovery of the molecular basis of magnetosome formation at a refined level. *M*. *magnetotacticum* MS-1 was the first magnetotactic bacterium to be isolated in pure culture [22]. However, MS-1 did not become a major model system for genetics since conditions that support colony growth on solid media have not been identified. Subsequently, AMB-1 and MSR-1 were successfully cultured and established as model organisms in the lab, making it possible to manipulate and investigate their genomes [23–25].

### Early studies of MTB genetics

The first genetic studies in MTB involved transposon mutagenesis coupled with magnetic selection and transmission electron microscopy (TEM). Matsunaga and colleagues (1992) performed mutagenesis in AMB-1 with the Tn5 transposon [23]. They identified several genomic fragments involved in magnetosome synthesis by picking out mutant cultures that no longer responded to a magnet under a light microscope. After confirming the mutants were deficient in magnetosome formation with electron microscopy, they used restriction mapping to narrow down the location of each insertion site in the genome. One of these mutants carries a transposon insertion in *magA*, a gene encoding a cation efflux pump proposed to function in iron transport [26]. Importantly, this study also demonstrated that it was possible to transfer plasmid DNA to AMB-1 using conjugation.

Large strides were made in understanding MTB genomes in the early 2000s. Wahyudi and colleagues (2001) also isolated Tn5 transposon mutants in AMB-1 and found colonies with defects in biomineralization by looking at colony color, which is thought to be an indicator of how much magnetite has accumulated in the cell [27]. They concluded that at least 10, and up to 60, genes could be involved in magnetosome formation. The publication of the genome sequences of MS-1 and *Magnetococcus marinus* MC-1 also opened the door to genome-level studies [28,29]. Grünberg and colleagues (2001) compared protein sequences isolated from MSR-1 magnetosomes to those in the MS-1 and MC-1 genomes and found two gene clusters containing genes (*mamA*, *mamB*, *mamC*, and *mamD*) we now know to be critical for magnetosome formation [30].

The key genomic region needed for magnetosome formation, the MAI, was discovered when spontaneous nonmagnetic mutants of MSR-1 were isolated from a wild-type population of cells [17]. It was characterized as a 130-kb region containing multiple insertion sequence (IS) elements [18]. The AMB-1 gene island was described as a 98-kb region flanked by two 1.1-kb repeat sequences [16]. A study of transcription of MAI genes indicated that while magnetosome genes are organized in operons, they are constitutively expressed [31]. Identifying the MAI narrowed down the pool of genes to investigate and provided a foundation for more targeted genetic studies.

**Fig 2:**
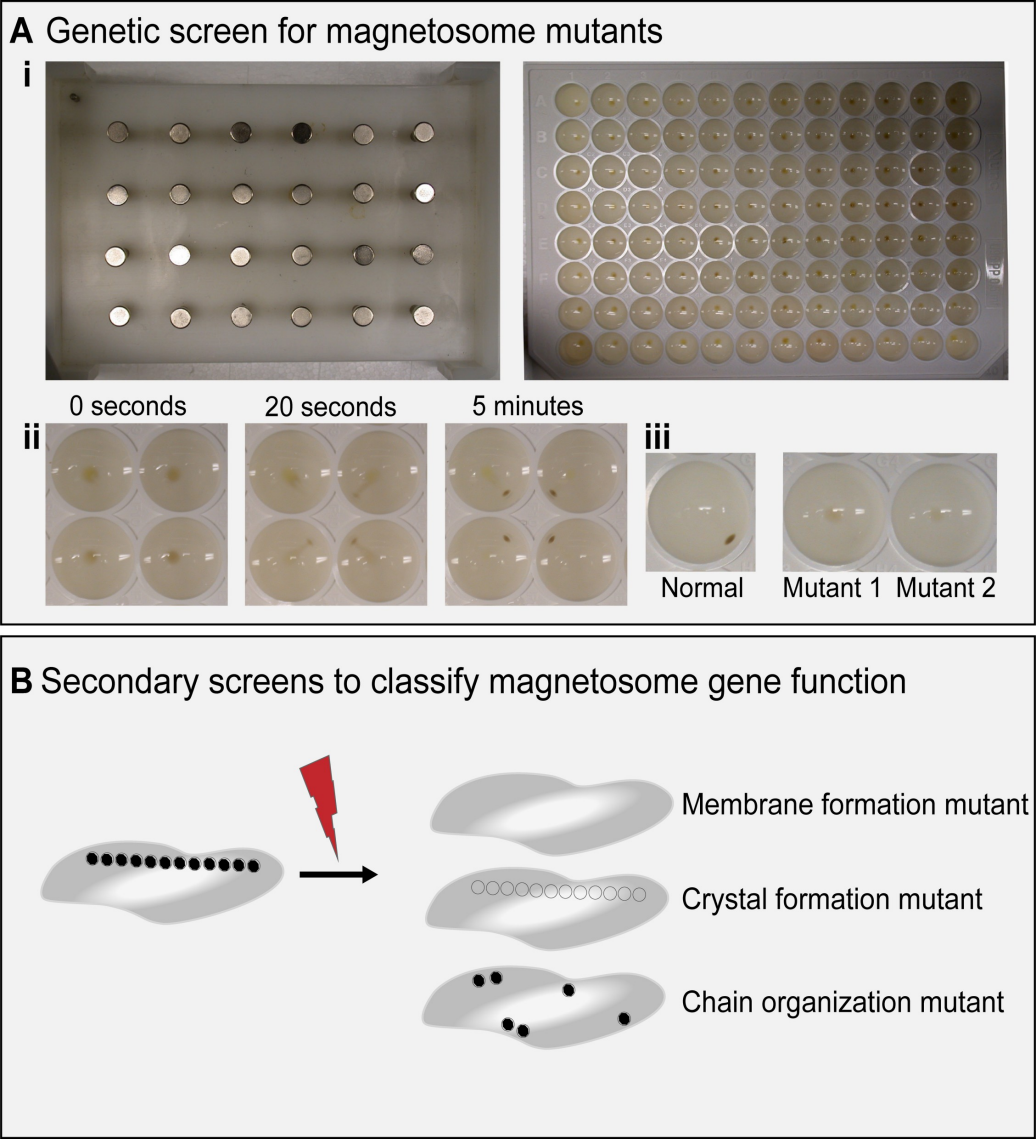
Magnetic screening technique. (A) (i) 24-pin magnetic plate (left) and 96-well plate of AMB-1 cells (right) used in Komeili and colleagues. [34]. (ii) Movement of AMB-1 cells on magnetic plate at 0 seconds, 20 seconds, and 5 minutes. (iii) Phenotype of normal magnetic cells (left) and two representative, nonmagnetic mutants (right). (B) Diagram of secondary screens to classify magnetosome mutants.

In addition to defining the MAI, the establishment of MSR-1 and AMB-1 as model systems allowed for more detailed molecular and genetic analyses [32,33]. A transposon mutagenesis screen by Komeili and colleagues (2004) used a magnetic selection to enrich for nonmagnetic mutants [34]. Colonies were then grown in 96-well plates and screened for magnetic response using a 24-pin magnetic plate (Fig 2A). In this study, transposon insertions within the *mamAB* gene cluster of the MAI resulted in nonmagnetic mutants. This work proved to be a great complement to proteomic studies that had found the same MAI-encoded proteins associated with magnetosomes [35].

Transposon mutagenesis studies proved to be a key turning point in using genetics to understand the process of biomineralization in MTB. However, much like many other genetic studies, their interpretation and broader utility were complicated by several confounding factors. First, due to homologous recombination between repeated sequences or potential action of transposases, the MAI is unstable and can be lost spontaneously, an event that is more likely to occur under stress conditions [18]. If transposition occurs in a bacterium that has lost its MAI or if the MAI is lost following transposition, otherwise neutral events may appear linked to changes in the magnetic phenotype. Screening potential mutants for the presence of the MAI proved essential in isolating mutations in the *mamAB* region [34].

Second, many magnetosome genes contain functional paralogs that play redundant roles. In AMB-1, three genes (*mamQ*, *mamR*, and, *mamB*) from the *mamAB* operon are perfectly duplicated in another segment of the MAI [36]. Additionally, the AMB-1 genome contains a magnetosome gene islet, a region outside of the MAI, which includes several homologs of *mamAB* genes [37,38]. As a result, the absence of a distinct phenotype when any of the duplicated genes are deleted individually does not rule out the possibility that they play a role in magnetosome formation. Thus, in many cases, multiple genes must be deleted to understand the function of a specific gene and its interactions with other genes. Additionally, complementing deletions becomes critical for the evaluation of gene function.

Third, magnetosome genes are often organized as operons and transposon insertions result in the polar loss of expression for all downstream genes. Thus, it is difficult to link a specific phenotype to the loss of one single gene. Finally, by necessity, these studies used the magnetic phenotype as a quick screening method to find relevant mutants. The secondary screens of transposon mutants were important for establishing which step in the magnetosome formation process was affected by a particular mutation and allowed for assigning more specific functions to genes (Fig 2B). For example, a nonmagnetic mutant might make magnetosome membranes but not form crystals, indicating the interrupted gene was likely involved in crystal formation. Or a nonmagnetic mutant might not make membranes at all, suggesting the site of transposon insertion is a key part of magnetosome membrane formation.

### Dissecting *Magnetospirillum* genomes

Obtaining the full genome sequences of the primary model organisms (MSR-1 and AMB-1) propelled genetic investigations of MTB forward [33,39]. Previous studies provided limited functional detail about any particular gene, at times involved looking at large deletions of multiple genes, and had complicating factors, as mentioned above. Deleting individual genes was necessary for a more complete picture of magnetosome formation.

Murat and colleagues (2010) used previously developed methodology to thoroughly dissect the MAI in AMB-1 by creating targeted deletions of genes and operons [36]. They began with the observation that the loss of the MAI results in complete absence of both magnetosome membranes and magnetic particles. Using a double recombination method for generating nonpolar deletions, they first made mutants lacking larger subsections of the MAI [34,36]. Next, they focused on the regions that showed dramatic phenotypes such as small particles or complete loss of the magnetosome membrane. Finally, they deleted individual genes within these flagged regions and used a suite of secondary screens to assign specific functions to the genes. Various electron microscopy techniques were used to visualize the magnetosome membrane as well as the size, morphology, and subcellular arrangement of magnetic particles. Green fluorescent protein (GFP) fusions to model magnetosome proteins were used to monitor protein localization.

Through multiple layers of analysis, Murat and colleagues described the possible functions of many of the key magnetosome-formation genes in AMB-1, like *mamE*, *mamN*, *mamM*, *mamO*, *mamI*, *mamL*, *mamQ*, and *mamB*. Lohße and colleagues (2011, 2014) dissected the MAI in MSR-1 [40,41] and found similar results, except that *mamI* and *mamN* were not essential for magnetosome formation in MSR-1. This discrepancy might be due to the particular growth conditions used obscuring more subtle differences between the two species. It may also reflect broader divergence between the two organisms. In a landmark study, heterologous expression of the *mamAB* and *mms6* operons, plus *mamGFDC* and *mamXYZ*, was found to be sufficient to produce magnetosomes in the nonmagnetic α-Proteobacterium *Rhodospirillum rubrum* [42], highlighting both the importance of these operons in magnetosome formation and the minimal gene set needed to make magnetosomes under laboratory conditions.

**Fig 3:**
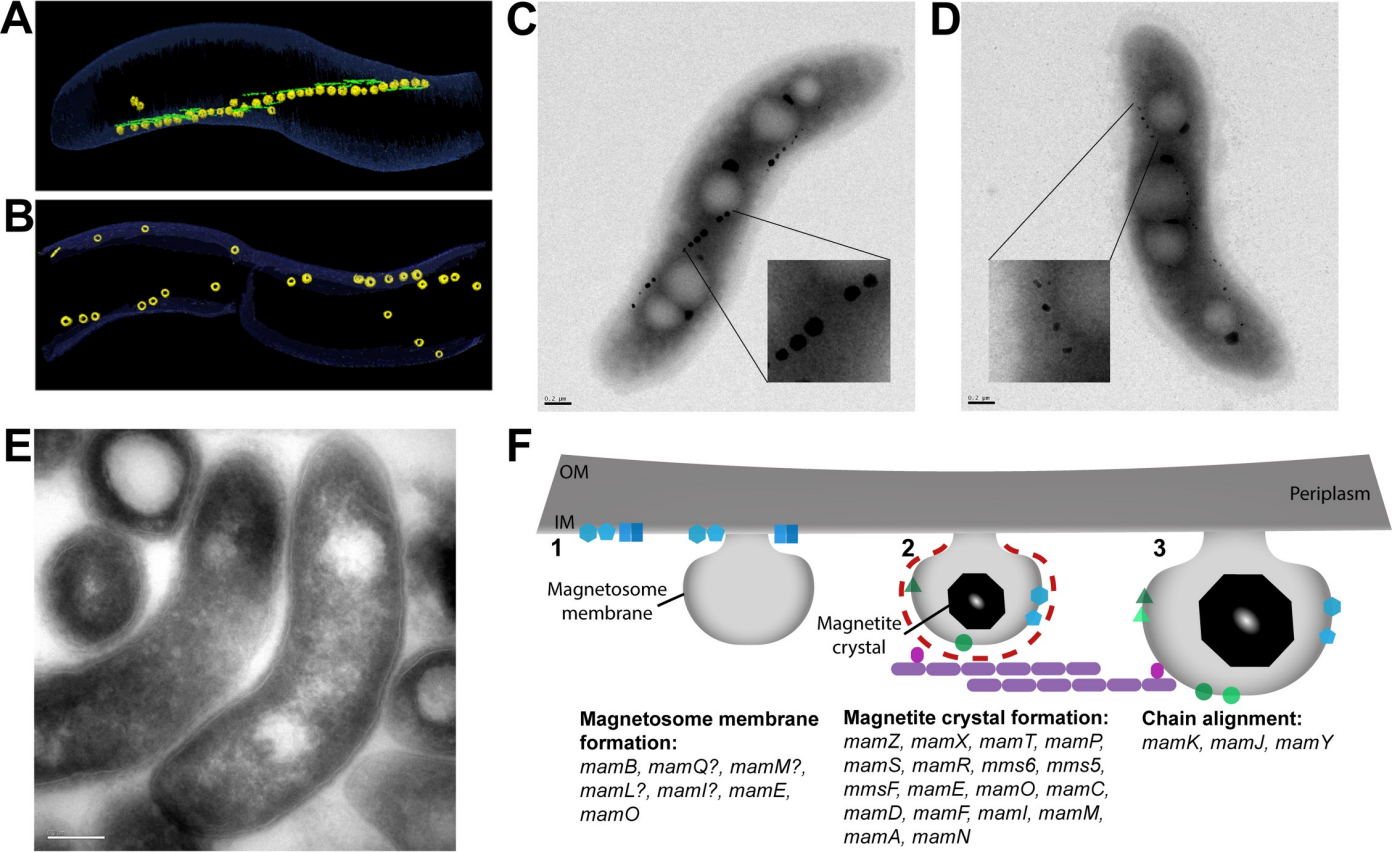
AMB-1 and MSR-1 strains with defects in magnetosome formation. (A) Wild-type, AMB-1–cell image taken from segmentation of an electron cryotomogram. MamK filaments (green) run parallel to magnetosomes (yellow). (B) Electron cryotomogram image of a Δ*mamK* AMB-1 cell that shows disorganized magnetosomes. Images provided by Komeili. (C) TEM image of a wild-type, AMB-1 cell. Scale bar is 0.2 μm. Close-up of magnetosomes is magnified 6×. (D) TEM image of a Δ*mamT* AMB-1 cell showing small, misshapen magnetosomes. Scale bar is 0.2 μm. Close-up of magnetosomes is magnified 6×. Image provided by McCausland and colleagues. (E) TEM image of a cryosection of Δ*mamL* AMB-1 cells showing that magnetosome membranes are absent. Scale bar is 0.2 μm. Image provided by Komeili. (F) Diagram of the stepwise process of magnetosome formation and the proteins involved from membrane invagination (1), to crystal nucleation (2), and to membrane growth and formation of a mature magnetic crystal (3). Genes that have been found to be involved at each step are listed. AMB-1, *Magnetospirillum magneticum* AMB-1; IM, inner membrane; OM, outermembrane; MSR-1, *Magnetospirillum gryphiswaldense* MSR-1; TEM, transmission electron microscopy.

These studies of the AMB-1 and MSR-1 islands provided a broad overview of the functions of genes in the MAI. Further studies dove into investigating the functions of individual genes in magnetosome formation and their mechanisms of action. The general framework that magnetosome genes are important for either membrane formation (Fig 3E) [43–45] or biomineralization (Fig 3C and 3D) [46–53] holds true. A third category of genes that are involved in chain organization has become an important area of study in recent years [38,54–59] (Fig 3A and 3B). At the center of the chain-arrangement process is an actin-like protein called MamK. Dynamic polymerization behavior of MamK is required for the integrity and proper segregation of the chain during cell division [54–58,60,61]. The proteins MamJ and MamY are also key components of chain formation. MamJ acts as a link between MamK filaments and magnetosomes [54]. MamY is a cytoplasmic membrane protein that works to align the magnetosome chain along the cell’s motility axis, which likely improves the efficiency of magnetotaxis [59]. To add nuance to the broad categories, more specific functions of genes involved in each stage of the process are being discovered as techniques and tools improve. More detailed summaries of the genes and proteins responsible for magnetosome formation can be found in previously published Review articles (Fig 3F) [62–64].

In addition to the limits of techniques used in genetic analysis, the growth conditions of MTB are an important factor to consider when examining the phenotype of particular genes. Genes both inside and outside the MAI have been found to have important roles in biomineralization but only under certain conditions. For example, within the MAI Δ*mamX*, Δ*mamZ*, Δ*mamH*, and Δ*ftsZm* strains only show defects in biomineralization when MSR-1 is grown with ammonium in place of nitrate, indicating that the use of oxygen instead of nitrate as the terminal electron acceptor is detrimental to magnetosome formation [65–67]. Genes outside the island, like the *nap* operon encoding nitrate-reductase genes [68] and the cytochrome *c* oxidase *cbb3* [69], are also key in the biomineralization process, again highlighting the importance of redox processes in magnetosome formation [70]. It is possible that these metabolic pathways generate an overall redox balance within the cell that is compatible with biomineralization, which requires both ferric and ferrous iron to be present. Alternatively, they may participate directly in generating a redox-balanced iron pool that will allow for magnetite biomineralization.

Another key environmental factor to consider in magnetosome formation is the availability of iron. Unsurprisingly, genes involved in regulating the uptake of iron have been connected to biomineralization. MSR-1 contains a homolog of the ferric uptake regulator (*fur*) gene common among bacteria [71]. This *fur*-like gene affects magnetosome size and number—potentially due to reduced incorporation of iron into magnetite and an increase in cytoplasmic iron concentrations—as well as transcription levels of several key MAI genes, including the *mamGFDC* and *mms6* operons [72]. Deletion of *feoB1*—which is involved in transport of ferrous iron into the cell—in MSR-1 resulted in fewer and smaller magnetosomes, as well as decreased uptake of iron [73]. In AMB-1, the *feoAB* operon showed increased levels of transcription under iron-rich conditions and was associated with an increase in intracellular iron levels, suggesting that it is a component of iron uptake in AMB-1 [74]. Ferric iron transporters were down-regulated in the same high-iron conditions. While the *feoAB1* operon is within the MAI, it is not a magnetosome-associated protein and thus its regulation of magnetosome formation is indirect. MS-1 also expresses *feoB*, indicating that iron uptake systems are conserved across species of MTB [75]. The iron response regulator IrrB was shown to be important for magnetosome formation in MSR-1, and deletion affected the transcription of several genes involved in the regulation of iron uptake [76]. These studies taken together indicate that iron availability is an important factor to consider in magnetosome formation and genetic regulation.

## Genetics and genomics of diverse MTB

As described above, work in the model organisms MSR-1 and AMB-1 has identified many of the genes that are important for the formation and positioning of magnetosomes. However, MSR-1 and AMB-1 are both α-Proteobacteria species and MTB are an incredibly diverse group of organisms with strains found in several classes of Proteobacteria, as well as Nitrospirae and the candidate division OP3 [3]. Little is known about the formation of magnetosomes in nonmodel organisms. The genetic studies in MSR-1 and AMB-1 have been used as jumping off points for the newer model system *D*. *magneticus* RS-1, as well as uncultured species of MTB like those from other classes of Proteobacteria and the phyla Nitrospirae [20]. Additionally, improved sequencing technologies have allowed the field to study the phylogeny of MTB using more relevant magnetosome genes instead of standard housekeeping genes.

### The MAI in *D*. *magneticus* RS-1

The establishment of the δ-Proteobacterium RS-1 as a model system has opened up the field to studying the genetic diversity of magnetosome formation. In the phylogeny of Proteobacteria, the δ-Proteobacteria class is deeply branching, relative to α-Proteobacteria. As such, research into δ-Proteobacteria can provide valuable insight into the origins of MTB. Through the study of RS-1, it has been found that, in addition to a core set of genes required across all MTB, different types of MTB have distinct genes for magnetosome formation. Presumably the genes evolved to adapt to the diverse lifestyles of each organism.

Comparative genome analysis of a variety of δ-Proteobacteria revealed that many of the genes required for magnetosome formation in α-Proteobacteria are shared by the δ-Proteobacteria, though some of the genes in the *mamAB* operon and the entire *mamGFDC* operon are missing [19]. It was discovered in the same study that the δ-Proteobacteria and Nitrospirae MTB have a separate set of class-specific genes termed the *mad* genes, which are likely involved in the production of bullet-shaped magnetite crystals. Additionally, Nitrospirae MTB have another set of genes, the *man* genes, that may be involved in the processes of magnetosome formation and/or chain arrangement that are particular to Nitrospirae [77]. The magnetosome gene sets that appear in different phyla provide a convincing link between genetic differences and the clear phenotypic differences seen across MTB. Model organisms from the δ-Proteobacteria and Nitrospirae are necessary to study the functions of *mad* and *man* genes.

In 1993, RS-1 was discovered as a sulfate-reducing MTB [78] and later identified as a *Desulfovibrio* species [79]. RS-1 is an obligate anaerobe that synthesizes bullet-shaped magnetite crystals, as opposed to the cubo-octahedral crystals produced by the α-Proteobacteria MTB species. While RS-1 was successfully cultured in the lab, attempts to delete individual genes were unsuccessful at first. Rahn-Lee and colleagues got around this problem with a classic forward genetic screen using random chemical and UV mutagenesis followed by whole-genome sequencing of nonmagnetic mutants of RS-1 [21]. Both *mam* genes and *mad* genes were found to be important in biomineralization, as were several novel MAI genes, including the ion transport genes *tauE* and *kup*. Genetic tools for deleting specific genes in RS-1 were only recently developed. Using suicide vectors for targeted gene deletion—as is commonly done in other bacterial systems—does not work in RS-1 due to low *trans*conjugation and recombination rates. Grant and colleagues developed a strategy using replicative plasmids that carry positive and negative selectable markers to replace the gene of interest with an antibiotic-resistance gene [80].

The study of magnetosome formation in RS-1 also identified a novel organelle consisting of iron–phosphorous granules surrounded by a membrane [81]. Byrne and colleagues showed that these granules are separate organelles and not precursors to the formation of magnetite with a pulse-chase experiment using different stable isotopes of iron. This conclusion was further solidified by the finding that the deletion of the entire MAI of RS-1 had no impact on the formation of the iron-rich granules [21]. The genetics of these novel bacterial organelles is a rich area for future investigation.

### The MAI in nonmodel and uncultured MTB

The genetic differences that have already been found between MSR-1, AMB-1, and RS-1 highlight the need to study diverse MTB species in order to more fully understand magnetosome formation and function(s). The rapid improvements in sequencing technologies in recent years—in addition to the elegant method for isolating MTB using an external magnetic field—have made it possible to study uncultured organisms in greater detail [82]. The genomes of many uncultured MTB have been sequenced and analyzed in detail [19,28,42,77,83–85], revealing that MTB belong to a wide variety of bacterial phyla. Metagenomic analyses are also contributing greatly to our knowledge of the diversity of MTB and their evolutionary history [10,20].

In the realm of nonmodel organisms, greigite-producing strains provide an interesting case to study the evolution of MTB. Work by DeLong and colleagues analyzing 16S rRNA gene sequences suggested that greigite-producing strains and magnetite-producing strains evolved separately [86]. However, a later analysis by Abreu and colleagues found that the greigite-producing strain *Candidatus* Magnetoglobus multicellularis has some of the *mam* genes that magnetite-producing strains require to produce magnetosomes, suggesting a monophyletic origin for MTB [87]. Lefèvre and colleagues (2011) discovered the δ-Proteobacteria *Desulfamplus magnetovallimortis* BW-1 and found that it is capable of producing both magnetite and greigite [85]. Interestingly, the BW-1 genome has *mam* genes in two separate MGCs. Proteins encoded in one cluster are closely related to proteins found in magnetite-producing species, while those in the second cluster are more closely related to the proteins encoded in the MGCs of greigite producers. The simplest hypothesis emerging from these genomic insights is that each cluster is responsible for producing a chemically distinct, magnetic mineral. Lefèvre and colleagues used the unique MGCs of BW-1 to examine phylogenetic differences between magnetite-producing and greigite-producing strains [19]. Genes required for producing magnetite-containing magnetosomes are clustered together, as are those required for producing greigite-containing magnetosomes, suggesting that there are separate sets of genes (and proteins) involved in forming each type of crystal. However, the *mad* genes, which are needed to form bullet-shaped magnetosomes, are present in both clusters. It is still unclear if *mad* genes were lost during the evolution of magnetite-producing strains that do not form bullet-shaped crystals or if they were acquired separately by δ-Proteobacteria and Nitrospirae strains of MTB. Analysis of the α-Proteobacteria PR-1 also indicated that evolution of MTB likely involved both vertical inheritance and horizontal gene transfer (HGT) or duplication events [84].

Looking further into the origins of MTB, Lefèvre and colleagues (2013) compared phylogenies of several α-, δ-, and γ-Proteobacteria and one Nitrospirae MTB species. They constructed phylogenetic trees using either 16S rRNA gene sequences and housekeeping genes or common Mam proteins [88]. They found that both trees showed a similar pattern of divergence, leading to the conclusion that all modern day Proteobacteria and Nitrospirae had a magnetotactic common ancestor, though they did not rule out the possibility of an ancient HGT event. Two recent studies from Lin and colleagues analyzed metagenomic data to gain insight into the origins of MTB [10,20]. The first study analyzed the genomes of multiple magnetotactic, Nitrospirae strains and found that the gene content and order in the MGCs were conserved across the Nitrospirae, indicating a common origin. In the second study, a wide variety of MTB genomes were analyzed using core magnetosome proteins and the phylogenetic trees showed MTB clustering together. The authors concluded, like Lefèvre and colleagues, that HGT of magnetosome genes were likely rare events. The simplest conclusion based on these studies is that all MTB originated from a common ancestor. In fact, using commonly accepted molecular clocks, it can be estimated that the original MTB—and presumably the first instance of magnetosome formation—appeared approximately 3.2 billion years ago [10]. An additional implication of this work is that at some point in the past the last common ancestor of the Proteobacteria, Nitrospirae, and Omnitrophica phyla had the genes necessary for formation of magnetic particles. The origins of magnetotactic Latescibacteria and Planctomycetes are less clear. These phyla could have emerged from the last common ancestor of the magnetotactic Proteobacteria, Nitrospirae, and Omnitrophica or acquired the genes through HGT. Subsequently, most descendants of these founding members lost the magnetosome genes leaving behind the handful of modern-day MTB. The environmental conditions and changes that initially favored the evolution and expansion of magnetosome-formation genes and later selected against them in the majority of bacteria remain to be elucidated. Perhaps, genetic studies of other model MTB are needed to understand the potential contributions of group-specific genes (such as *mad* and *man* genes) to the evolution and phenotypic diversification of magnetosomes.

The study of uncultured MTB has also been aided through the analysis of gene function in model MTB. Take, for example, the MAI genes *mamE* and *mamO*, which are critical for biomineralization in both AMB-1 and MSR-1 [36,89]. Both gene products are predicted serine proteases, and initial genetic studies concerning their functions concluded that this was indeed the case [90]. However, further biochemical and structural studies revealed that the active site of MamO is not functional and that it is in reality a metal-binding protein that controls biomineralization and regulates the proteolytic activity of MamE [91,92]. Phylogenetic analyses showed that, similar to AMB-1, all proteobacterial MTB encode an active and inactive protease in their MGCs [91]. The active protease is ancestral to all MTB and has been diversified through vertical descent. However, the inactive protease has arisen multiple times in MTB through duplications of the active protease or acquisition via HGT. These insights were only possible through a combination of genetic, genomic, and biochemical studies. They highlight the critical analyses needed in studies in which duplication events and diversification of function of similar proteins can blur the accuracy of phylogenetic studies. They also show that the study of a protein in one species of MTB may not clarify the function of a homologous protein in another related organism.

## Outlook

Huge strides have been made in understanding the genetics behind magnetosome formation. While the minimum set of genes required to generate magnetosomes is known, the specific roles of many of these factors remain unknown. Additionally, there are several genes within the MAI that when deleted have subtle or not obvious phenotypes. There are also presumably many genes outside the island that have key, though indirect, roles in magnetosome formation.

**Fig 4:**
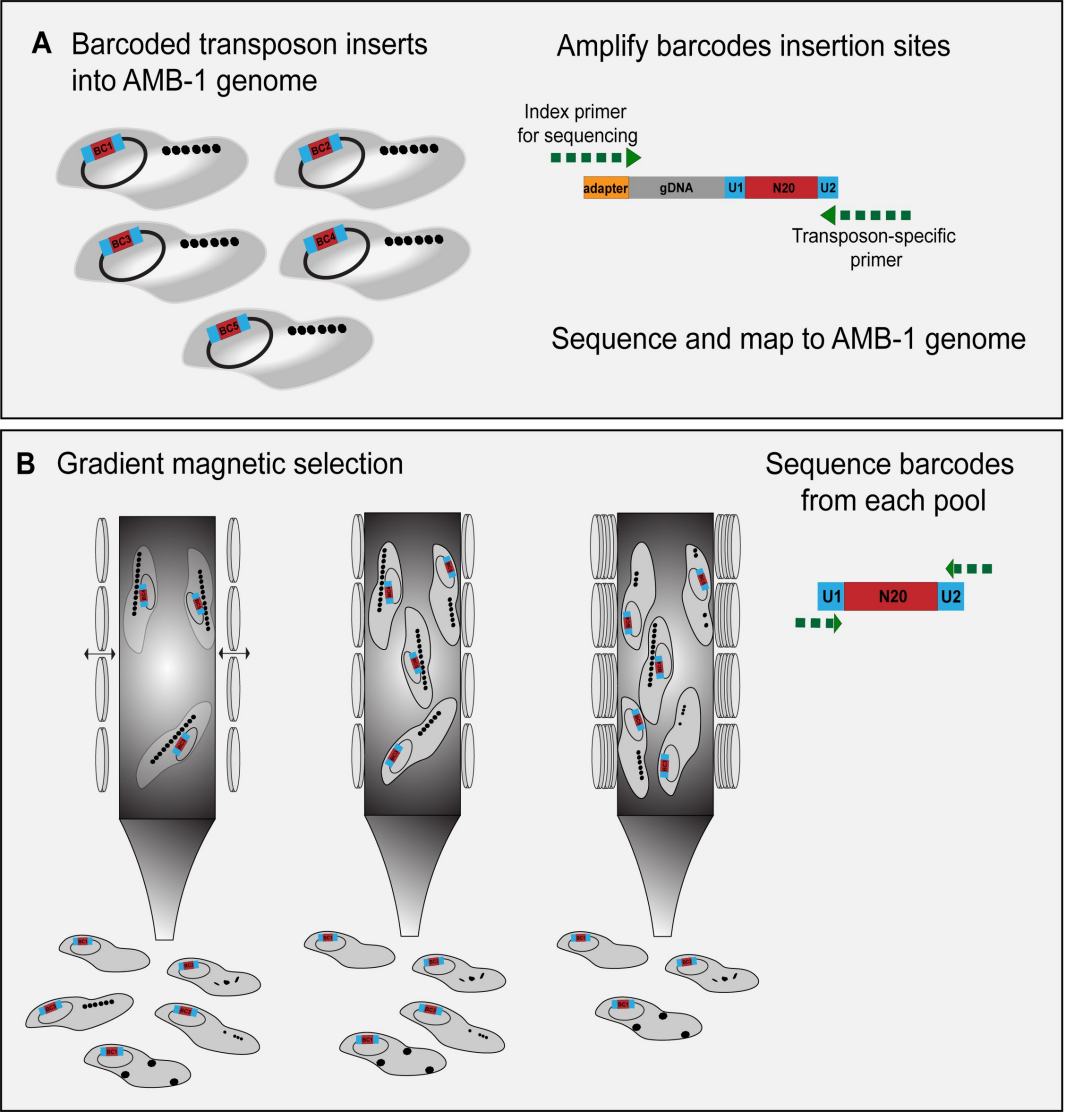
Barcoded transposon mutagenesis and a potential magnetic screen. (A) Diagram of RB-TnSeq in AMB-1. Each transposon insertion carries a unique 20-nucleotide sequence that acts as a barcode. The mutated strains are pooled, and then the barcodes are mapped to their insertion site in the genome. (B) Diagram of magnetic selection with the TnSeq library using different magnetic strengths to select for a range of mutant phenotypes. From left to right, as magnetic strength applied to the column increases, strains with weaker magnetic responses will be able to stick to the column, yielding a gradient of magnetic phenotypes to analyze. AMB-1, *Magnetospirillum magneticum* AMB-1; BC1, barcode inserted into AMB-1 genome; gDNA, genomic DNA; N20, unique 20-nucleotide sequence; RB-TnSeq, random barcoded transposon-site sequencing; U1, universal polymerase chain reaction priming site.

Genetic screens provide a high-throughput strategy for uncovering novel genes. Transposon mutagenesis, in particular, has been used multiple times to study the genomes of AMB-1 and MSR-1. In the future, we envision several improvements that can make transposon mutagenesis an even more useful method for genetic investigation of MTB. The latest techniques in transposon mutagenesis involve pooling tens to hundreds of thousands of labeled mutants with a method called random barcoded transposon-site sequencing (RB-TnSeq) [93] (Fig 4A). This strategy allows for saturated coverage of a bacterial genome and averaged impact of gene loss across multiple mutants. As such, polar effects and individual off-target effects are minimized during phenotyping. However, genes identified in transposon-mutagenesis screens still require phenotypic validation with gene deletions. The development of more advanced gene-editing technologies, like CRISPR, will be just as valuable for the study of MTB as it has been for other organisms [80,94]. For example, CRISPR interference (CRISPRi) can be used to knockdown multiple genes at a time more readily than traditional methods or to study essential genes by tuning their expression [95,96]. Additionally, stepping aside from transposon mutagenesis and screening instead for point mutants that have conditional phenotypes, act as dominant alleles, or suppress known mutant phenotypes can help to expand our understanding of the genetic networks that participate in magnetosome formation.

The methods for screening after the initial mutagenesis can also be refined. Screens thus far have mostly relied on a simple binary identification of magnetic versus nonmagnetic cells or have used colony color as a proxy for magnetite formation. The ability to identify mutants on a spectrum of magnetic responses would be highly informative for understanding the process of magnetosome formation (Fig 4B). It may be possible to use microfluidics for this approach [97,98]. Methods that simply allow for the capture of more magnetic mutants are also key to saturate screens and identify potential negative regulators of magnetosome formation.

Screening methods that allow for the identification of genes with more subtle phenotypes on a spectrum will open the field to studying both genes in the MAI previously thought to have little to no effect on magnetosome formation or genes outside the MAI that are key in magnetosome formation under specific growth conditions. Multiple genes outside the MAI—primarily involved in metabolism—have already been connected to magnetosome formation. For example, the nitrate-reductase genes of the *nap* operon are important for magnetosome formation in MSR-1, even when oxygen is available as the terminal electron acceptor [68]. And the metabolic regulator *crp* has also been tied to magnetosome formation [99]. Different species of MTB migrate to a variety of preferred oxygen concentrations (all under 25 μM) using one of three patterns of magneto-aerotaxis [100]. The genetic mechanisms behind aerotactic behavior have begun to be investigated. For example, Popp and colleagues showed that the chemotaxis operon *cheOp1* was necessary for the aerotactic response in MSR-1 [101].

The insights into metabolism and magnetosome formation are naturally connected to the increasing interest in the field in studying the ecological role of MTB in their natural environments [102–104]. How MTB interact with and adapt to changing conditions will also be an interesting problem from the perspective of geneticists and cell biologists. Most studies on MTB have been done under tightly controlled laboratory conditions, but in nature, MTB encounter changes in pH, temperature, oxygen gradients, and nutrient levels. A Review by Moisescu and colleagues summarizes the effects of these changes on magnetosome formation [105]. In addition, the study of environmental conditions may help us understand how extremophile MTB evolved or retained the ability to form magnetosomes in conditions that are not viable for most MTB species [106].

## Conclusion

The set of genes needed for magnetosome formation has been clearly determined across multiple organisms, and many of their functions have been investigated. However, it is clear that even in the most well-studied MTB, like AMB-1 and MSR-1, the roles of genes that work in tandem with other factors, that participate in multiple aspects of magnetosome formation, or that are only required conditionally have yet to be fully understood. Going forward, more nuanced study of genes involved in magnetosome formation will be key to expanding our knowledge of MTB for basic cell biology, ecology, and biotechnology applications. Additionally, the study of diverse MTB using both targeted genetic analyses and whole-genome studies will potentially clarify the functions of many genes, while also adding layers to our picture of MTB.
